# Structural basis for DAXX interaction with ATRX

**DOI:** 10.1007/s13238-017-0462-y

**Published:** 2017-09-05

**Authors:** Xiaoman Wang, Yiyue Zhao, Jian Zhang, Yong Chen

**Affiliations:** 1 State Key Laboratory of Molecular Biology, National Center for Protein Science Shanghai, Shanghai Science Research Center, CAS Center for Excellence in Molecular Cell Science, Shanghai Institute of Biochemistry and Cell Biology, Chinese Academy of Sciences, University of Chinese Academy of Sciences, Shanghai, 201210 China; 2 School of Life Science and Technology, Shanghai Tech University, Shanghai, 201210 China

**Dear Editor**,

Alpha-thalassemia/mental retardation syndrome X-linked protein (ATRX) is a member of the switch 2/sucrose nonfermentable 2 (SWI2/SNF2) family of chromatin-remodeling proteins (Clynes et al., 2013; Dyer et al., 2017). ATRX deposits histone variant H3.3 into heterochromatin loci with the cooperation of an H3.3-specific chaperone, the death-domain associated protein (DAXX) (Goldberg et al., 2010; Law et al., 2010; Lewis et al., 2010). Loss of ATRX or DAXX leads to an increased DNA damage response, activation of the alternative lengthening of telomeres (ALT) pathway, and genomic instability (Dyer et al., 2017). Consequently, genome sequencings have identified ATRX and DAXX mutations in a variety of cancers (Watson et al., 2015). Due to the important roles of the DAXX-ATRX complex in the maintenance of heterochromatin structure and stability, the structural studies of ATRX and DAXX have been extensively carried out.

ATRX contains two structural domains. One is the N-terminal ADD (ATRX-DNMT3-DNMT3L) domain that specifically recognizes H3 lysine 9 trimethylation (H3K9me3) (Iwase et al., 2011). The other one is C-terminal ATP-dependent chromatin-remodeling domain, which has not been structurally characterized. DAXX also contains two structural regions. One is N-terminal DAXX helical bundle (DHB) domain, which has been shown to interact with RASSF1C (Ras-association domain family 1 isoform C), P53 and MDM2 (mouse double minutes 2 homolog) (Escobar-Cabrera et al., 2010). The other one is histone binding domain (HBD), responsible for specific recognition of H3.3-H4 (Elsasser et al., 2012; Liu et al., 2012). However, the manner in which DAXX interacts with ATRX to orchestrate the histone chaperone activity of DAXX and the chromatin remodeling activity of ATRX remains largely unclear.

In the present study, we first dissected the interaction between DAXX and ATRX. The DAXX helical bundle (DHB) domain has been shown to interact with two modules of ATRX (Tang et al., 2004). Residues between 1,189 and 1,326 of ATRX serve as the dominant binding module for DAXX, and residues 321–865 of ATRX may constitute a secondary DAXX-interacting module (Tang et al., 2004). Here we used isothermal titration calorimetry (ITC) to evaluate the contribution of each ATRX module to the DAXX-ATRX interaction. We found that ATRX_1,188–1,326_ interacts with DAXX_DHB_ with a *K*_d_ of 160 nmol/L, while ATRX_321–866_ undergoes no detectable binding to DAXX_DHB_ (Fig. S1A). Therefore, we focused on ATRX_1,188–1,326_ for further investigation. We generated a panel of ATRX fragments spanning 1,188–1,326 and examined their binding capacities with DAXX_DHB_ (Fig. S1B). A minimal ATRX fragment consisting of residues 1,260–1,289 was both necessary and sufficient to interact with DAXX_DHB_ (Fig. S1B and S1C). Hereafter, we will refer to ATRX_1,260–1,289_ as the DAXX-binding motif of ATRX (ATRX_DBM_) (Fig. 1A).

**Figure 1:**
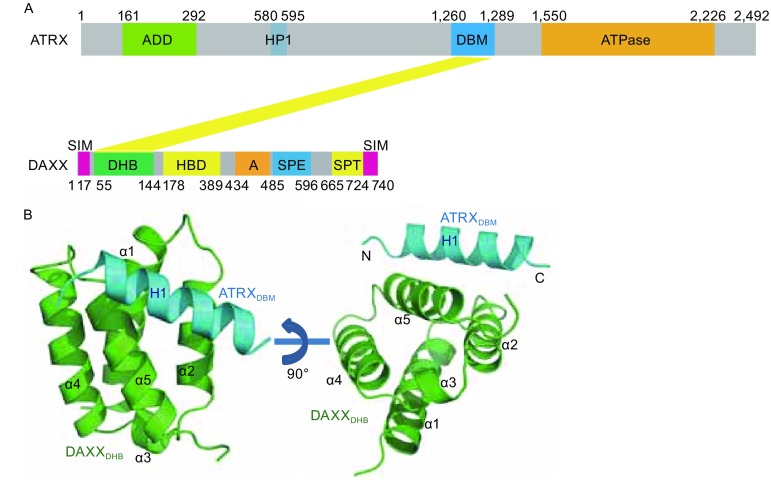
**The structure of the DAXX**_**DHB**_ **-ATRX**_**DBM**_ **complex**. (A) Domain organization of the ATRX and DAXX. ADD, ATRX-DNMT1-DNMT1L domain; HP1, HP1-binding motif; DBM, DAXX binding motif; ATPase, ATPase domain; SIM, Sumo-interaction motif; DHB, DAXX helical bundle; HBD, histone binding domain; Acidic, segment rich in Glu/Asp residues; SPE, segment rich in Ser/Pro/Glu residues; SPT, segment rich in Ser/Pro/Thr residues. (B) Two orthogonal views of the DAXX_DHB_-ATRX_DBM_ complex. DAXX_DHB_ is colored in green and ATRX_DBM_ is colored in cyan

We determined the structure of the DAXX_DHB_–ATRX_DBM_ complex at a resolution of 2.2 Å using single-wavelength anomalous dispersion with selenomethionine-substituted crystals (Table S1). The structure has been refined to an *R*-value of 18.7% (*R*_free_ = 21.9%) with good geometry. The electron density map allowed us to trace most of the complex without much ambiguity (Fig. S2A). The final refined model covered DAXX residues 57–141 and ATRX residues 1,267–1,284. DAXX_DHB_ forms an elongated helix bundle with four antiparallel packed helices α1, α2, α4, and α5 (Fig. 1B). α3 is a short helix connecting α2 and α4, and it crosses the base of the helical bundle. ATRX_DBM_ exists as a long amphipathic helix (residues 1,269–1,283) lying along the cleft between helices α2 and α5 of DAXX_DHB_ (Fig. 1B). ATRX_DBM_ binding does not induce large conformational change in DAXX_DHB_, because the DAXX_DHB_ structure in the complex is almost identical to the previously determined NMR structure of apo DAXX_DHB_ (Escobar-Cabrera et al., 2010), with a root-main-square deviation (rmsd) value of 1.0 Å for 83 equivalent Cα pairs.

The interaction between DAXX_DHB_ and ATRX_DBM_ is predominantly mediated by hydrophobic contacts. Four nonpolar residues (A1272, L1276, L1277, and I1280) in the center of the ATRX_DBM_ helix constitute a hydrophobic core that fits snugly into a shallow groove in DAXX_DHB_ (Fig. 2A). The side chains of these residues make close contacts with a panel of hydrophobic amino acids, including V84, F87, Y124, V125, and I127 of DAXX (Fig. 2A). Consistent with the structural model, mutations of any of the hydrophobic residues on ATRX destabilized the DAXX_DHB_-ATRX_DBM_ interaction (Fig. 2B). In particular, mutations in ATRX ^L1276^ and ATRX^L1280^ had the most disruptive effects, and double mutants (ATRX^L1276R/L1280R^ and ATRX^L1276Q/L1280Q^) completely abolished the interaction with DAXX_DHB_ (Fig. 2B). Similarly, the hydrophobic residues on DAXX_DHB_ are also crucial to the binding of ATRX (Fig. 2C). Point mutations of these hydrophobic residues impaired the DAXX_DHB_-ATRX_DBM_ interactions, and a DAXX double mutant (F87A/Y124A) completely lost its ability to bind to ATRX (Fig. 2C). These data indicate that the hydrophobic interactions are the major driving force for the binding of ATRX_DBM_ to DAXX_DHB_. These hydrophobic residues in DAXX and ATRX are well conserved across many species (Fig. S2B and S2C), suggesting that DAXX-ATRX in other species may also adopt the same interaction mode. The only exception is the *Drosophila* counterparts of DAXX and ATRX, dDAXX and dXNP. For example, DAXX F87 is replaced with a glutamate in dDAXX, and ATRX I1280 by an arginine in dXNP (Fig. S2B and S2C). These differences may severely impair the interaction between dXNP and dDAXX. Whether and how the *Drosophila* counterparts interact with each other remains to be determined.

**Figure 2:**
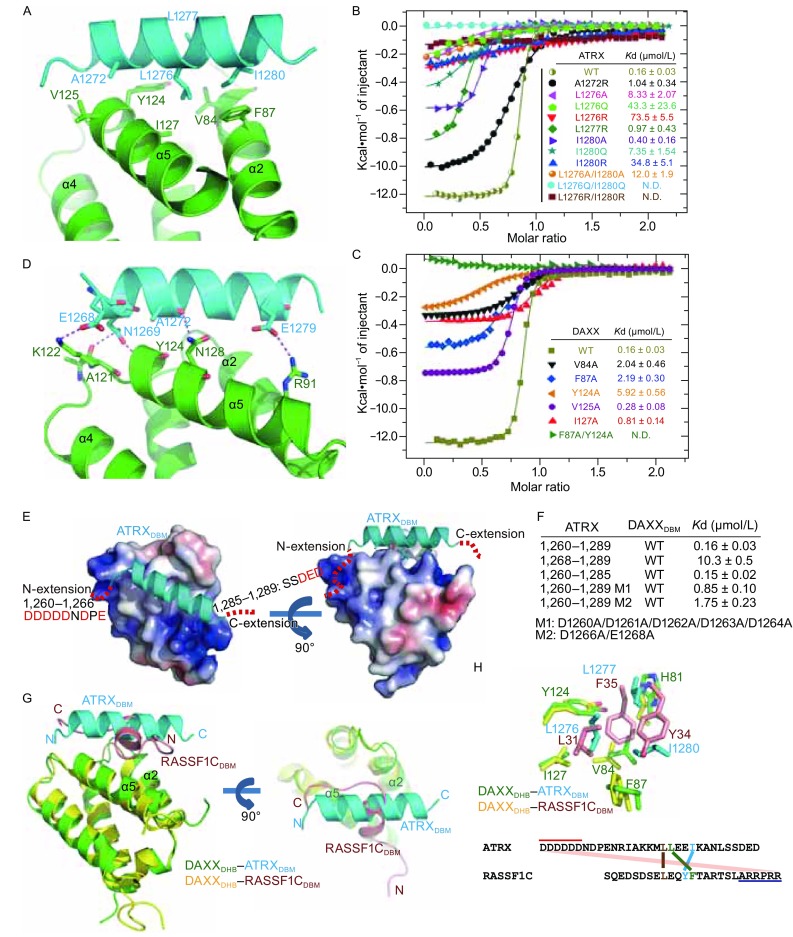
**The interfaces between DAXX**_**DHB**_ **and ATRX**_**DBM**_. (A) Details of hydrophobic contacts in the DAXX_DHB_-ATRX_DBM_ interface. The contacting residues are presented as ball-and-stick models. DAXX residues are colored in green and ATRX residues are colored in cyan. (B and C) Effects of mutations in the ATRX_1,253–1,326_ (B) and DAXX_55–144_ (C) on the interaction between DAXX and ATRX analyzed by ITC assays. The *K*_d_ values for WT and mutants were indicated. “N.D.” stands for “Not Detectable”. (D) Details of salt bridge and hydrogen-bonding interactions between DAXX_DHB_ and ATRX_DBM_, shown as dashed red lines. (E) The N- and C-terminal extensions of ATRX_DBM_ may interact with DAXX_DHB_. DAXX_DHB_ is shown in surface representation and colored according to its electrostatic potential (positive potential, blue; negative potential, red). The absent N- and C-terminal extensions cannot be modeled unambiguously. The red dashed lines indicate possible location of these missing extensions for illustration purpose. (F) Effects of truncations and mutations of ATRX_DBM_ on DAXX-ATRX interactions shown by ITC assays. (G) Superimposition of DAXX_DHB_-ATRX_DBM_ and DAXX_DHB_-RASSF1C_DBM_ (PDB: 2KZU) structures shown in two orthogonal views. DAXX_DHB_ in DAXX_DHB_-ATRX_DBM_ complex, green; ATRX_DBM_, cyan; DAXX_DHB_ in DAXX_DHB_-RASSF1C_DBM_ complex, yellow; RASSF1C_DBM_, red. (H) The conserved hydrophobic interfaces in two structures. The structural equivalent residues are indicated. (I) The sequence alignment of ATRX_DBM_ and RASSF1C_DBM_ shows low similarity. The structural equivalent residues are not sequentially aligned

Complementary with the hydrophobic contacts, a series of salt bridges and hydrogen-bonding interactions further strengthened the interactions between DAXX_DHB_ and ATRX_DBM_. The carboxylate group of ATRX^E1268^ engages in a salt-bridge interaction with DAXX^K122^, while ATRX^E1279^ coordinates a salt bridge with DAXX^R91^ (Fig. 2D). In addition, the carboxamide group of ATRX^N1269^ forms two hydrogen bonds with the backbone carbonyl of DAXX^A121^ and the backbone amide of DAXX^Y124^ (Fig. 2D). The carbonyl of ATRX^A1272^ forms a hydrogen bond to DAXX^N128^ (Fig. 2D). In addition to these polar interactions observed in the structure, the N- and C-terminal extensions of ATRX_DBM_ may also contribute to binding with DAXX_DHB_ through electrostatic interactions. Calculation of the electrostatic potential of DAXX_DHB_ shows that the amphipathic helix of ATRX_DBM_ is clamped by two basic patches of DAXX (Fig. 2E). Correspondingly, the N- and C-terminal extensions of ATRX_DBM_ are rich in acidic residues (Fig. 2E). Although the N- and C-terminal extensions are absent from the current structure, the close spatial disposition of these acidic extensions of ATRX_DBM_ and basic patches of DAXX_DHB_ strongly suggest that the acidic regions of ATRX_DBM_ are associated with DAXX_DHB_ through electrostatic interactions. To investigate the roles of these extensions, the effect of N- or C-terminal truncation of ATRX_DBM_ was examined by ITC assays. Although C-terminal truncation had no effect on DAXX_DHB_-ATRX_DBM_ interaction, deletion of N-terminal eight residues resulted in a ~60-fold decrease in the affinity between DAXX_DHB_ and ATRX_DBM_ (Fig. 2F), indicating that the N-terminal extension is essential for strong binding between DAXX_DHB_ and ATRX_DBM_. Mutations of acidic residues in the N-terminal extension of ATRX_DBM_ also weakened the interaction between DAXX_DHB_ and ATRX_DBM_ (Fig. 2F), further underscoring the importance of the N-terminal-extension-mediated electrostatic interactions. Taken together, these extensive hydrophobic, electrostatic, and hydrogen-bonding interactions ensure a stable association between DAXX and ATRX.

DAXX is a scaffold protein that interacts with more than 50 proteins with diverse roles (Lindsay et al., 2008). The DAXX helical bundle (DHB) domain has been reported to interact with ATRX, RASSF1C, MDM2, HAUSP, P53, P63, and P73 (Escobar-Cabrera et al., 2010; Gostissa et al., 2004; Tang et al., 2006; Tang et al., 2004). The molecular mechanism by which DAXX_DHB_ recognizes different partners remains poorly understood. Here we compared complex structures of DAXX_DHB_-ATRX_DBM_ and DAXX_DHB_-RASSF1C_DBM_. ATRX_DBM_ and RASSF1C_DBM_ both exist as amphipathic helices and bind to the same groove between helices α2 and α5 of DAXX_DHB_ (Fig. 2G). Although these two DBMs show low sequence homology, key residues involved in hydrophobic contacts are conserved (Fig. 2H and 2I). ATRX L1276, L1277, and I1280 occupy positions corresponding to those of L31, F35, Y34 of RASSF1C, respectively (Fig. 2H). These structural equivalent residues interact with the same panel of hydrophobic residues of DAXX (Fig. 2H). Notwithstanding these similarities, there are substantial structural differences between ATRX_DBM_ and RASSF1C_DBM_. First, they exhibit distinct orientations within the complex structures. ATRX_DBM_ extends across the α2 and α5, while RASSF1C_DBM_ is anti-parallel to α2 and α5 of DAXX_DHB_ (Fig. 2G). In this way, these two DBM helices are almost perpendicular to each other. Second, both N- and C-terminal extensions of ATRX_DBM_ are acidic in nature, while RASSF1C_DBM_ has a negatively charged N-terminal extension and a positively charged C-terminal tail. Due to the topological difference between ATRX_DBM_ and RASSF1C_DBM_, the basic C-terminal tail of RASSF1C_DBM_ is close to the basic patch of DAXX_DHB,_ which is where the acidic N-terminal extension of ATRX_DBM_ binds (Fig. 2E and 2G). The basic C-terminal tail of RASSF1C_DBM_ may interfere with the otherwise strong binding to DAXX_DHB_. This may explain the relatively low binding affinity between DAXX_DHB_ and RASSF1C_DBM_ (*K*_d_ = 65 μmol/L) (Escobar-Cabrera et al., 2010).

In summary, the structural characterization of the DAXX_DHB_ domain in complex with ATRX_DBM_ provides a molecular framework for understanding the interaction between DAXX and ATRX. The DAXX-ATRX interaction is a crucial link to bridge the chaperone-activity domain of DAXX and the remodeling-activity domain of ATRX together to deposit H3.3 into heterochromatin foci. The structural model and mutagenesis data presented here also provide an opportunity to dissect the functional consequences of specific disruption of DAXX-ATRX *in vivo*. Although there are a few of disease mutations identified in regions of ATRX_DBM_ and DAXX_DHB_, none of these mutations seems to affect DAXX-ATRX interaction (Fig. S3). Why the DAXX-ATRX interface is not susceptible to disease mutations needs further investigation. Moreover, our structural analyses of DAXX_DHB_-ATRX_DBM_ and DAXX_DHB_-RASSF1C_DBM_ indicate that DAXX_DHB_ is a general protein-interaction domain with sufficient structural plasticity to accommodate DBMs from different interaction partners. Given that the topological relationships of these DBMs are completely different, at this stage, it would be difficult to detect the hidden similarities among these DBMs based solely on sequence information, without 3D structural information. As more DAXX_DHB_-interaction partners are identified and their structures become available, it should be possible to identify the conserved features of these interaction partners in the future.

## Electronic supplementary material

Below is the link to the electronic supplementary material.

Supplementary material 1 (PDF 749 kb)
