# Supplementary material for: Structural basis for DAXX interaction with ATRX
Source: Protein Cell. 2017 Sep 5;8(10):767–71. doi: 10.1007/s13238-017-0462-y (PMC5636753; doi:10.1007/s13238-017-0462-y)
Supplement: Supplementary file 1 — Supplementary material 1 (PDF 749 kb) [file 13238_2017_462_MOESM1_ESM.pdf]

Methods

Protein expression and purification

All the ATRX (Uniprot: P46100) constructs were cloned into a modified pET-28b vector with a His_6-SUMO tag fused at the N terminus. All the DAXX (Uniprot: Q9UER7) constructs were cloned into a PGEX-6P vector containing a GST tag followed by a 3C protease site. *Escherichia coli* Rosetta cells bearing expression plasmids were induced for 16 h with 0.1 mM IPTG at 18 ℃, and the cells were collected by centrifugation. The cell pellets were resuspended in lysis buffer (50 mM Tris-HCl, pH 8.0, 400 mM NaCl, 10% glycerol, 2 mM 2-mercaptoethanol, and home-made protease inhibitor cocktail). The cells were broken by sonication and cleared by ultracentrifugation at 100,000*g* for 30 min. The proteins were purified using Ni-NTA agarose beads (Qiagen) for His_6-Sumo-tagged proteins or Glutathione sepharose 4B beads (GE Healthcare) for GST-tagged proteins, followed by ULP1 and 3C protease digestion respectively to remove the tags. The proteins were further purified by gel-filtration chromatography on Hiload Superdex 75 column (GE Healthcare) equilibrated with buffer (25 mM Tris-HCl pH 8.0, 150 mM NaCl). The purified proteins were concentrated to 10–25 mg ml^{-1} and store at −80 °C. Mutations were introduced by PCR-based site-directed mutagenesis, and mutated proteins were purified using the same protocol as described above.

For purification of $DAXX_{DHB}$-$ATRX_{DBM}$ complex, the cell pellets expressing GST-$DAXX_{DHB}$ and Sumo-$ATRX_{DBM}$ were mixed and purified by tandem affinity columns (Ni-NTA agarose beads and glutathione sepharose beads). Ulp1 and 3C proteases were

added to remove the His_6-Sumo and GST tags, respectively. The complex was further purified by gel-filtration chromatography. The SeMet-substituted complex was similarly purified.

Crystallization, data collection and structural determination

For structural studies, numerous combinations of different ATRX and DAXX fragments were screened for crystallization. Although most of the constructs can be crystallized, they are not amendable for high-resolution diffraction. The complex suitable for high-resolution structural determination is composed of $DAXX_{55\text{-}144}$ and ATRX $_{1267\text{-}1289}$. The $DAXX_{55\text{-}144}$—$ATRX_{1267\text{-}1289}$ complex was crystallized in 0.2M ammonium acetate, 0.1M sodium citrate, pH5.6, 30% PEG4000 at 16 ℃. All crystals were gradually transferred into harvesting solutions (precipitant solution with 22% glycerol) before being flash-frozen in liquid nitrogen. SeMet-SAD diffraction data was collected at the beamline BL19U1 of the Shanghai Synchrotron Radiation Facility with a wavelength of 0.9785 Å at 100 K, and processed using HKL3000 (Minor et al., 2006). Selenium sites finding, SAD phases calculation, solvent-flattening density modification and automatic model building were done by AutoSHARP (Vonrhein et al., 2007). The model was then refined by PHENIX package (Adams et al., 2002) with manual rebuilding in COOT (Emsley and Cowtan, 2004).

Isothermal titration calorimetry

The equilibrium dissociation constants of DAXX-ATRX interactions were determined by an MicroCal ITC200 calorimeter (Malvern). The binding of DAXX protein (0.5–2mM)

and ATRX protein (30–200 μM) were measure in the buffer (25 mM Tris-HCl, pH 8.0, 150 mM NaCl) unless stated at 20 ℃. ITC data were analyzed and fit in Origin 7 (OriginLab) using one-site model.

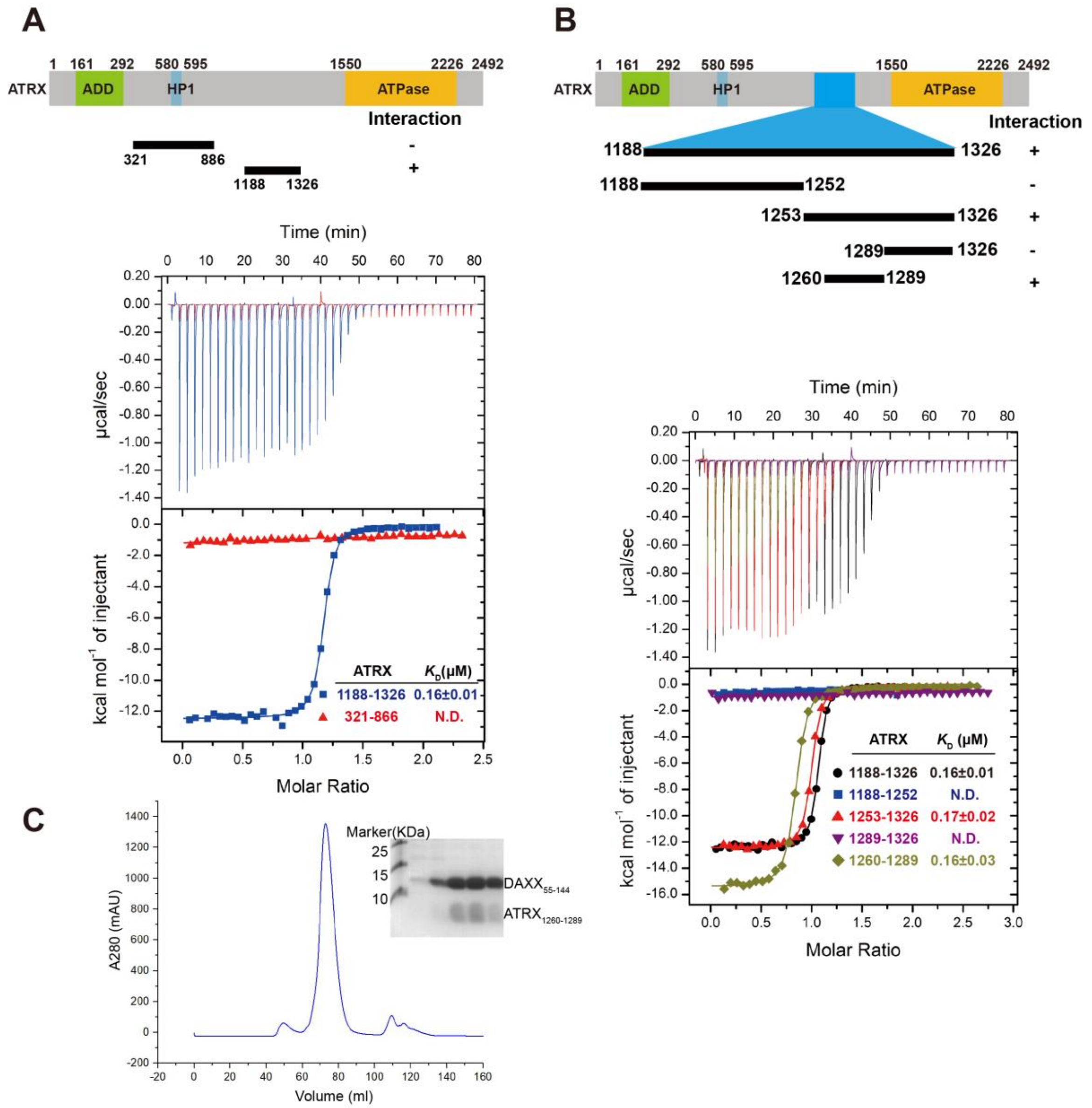

Supplementary Figure 1

Supplementary Figure 1. Mapping interaction domains between DAXX and ATRX.

A. Evaluation of contributions of two previous reported ATRX fragments to DAXX-ATRX interaction by ITC assays.

B. ITC assays identified the minimum DAXX-binding motif of ATRX.

C. Purification of the binary complex composed of $DAXX_{DHB}$ and $ATRX_{DBM}$.

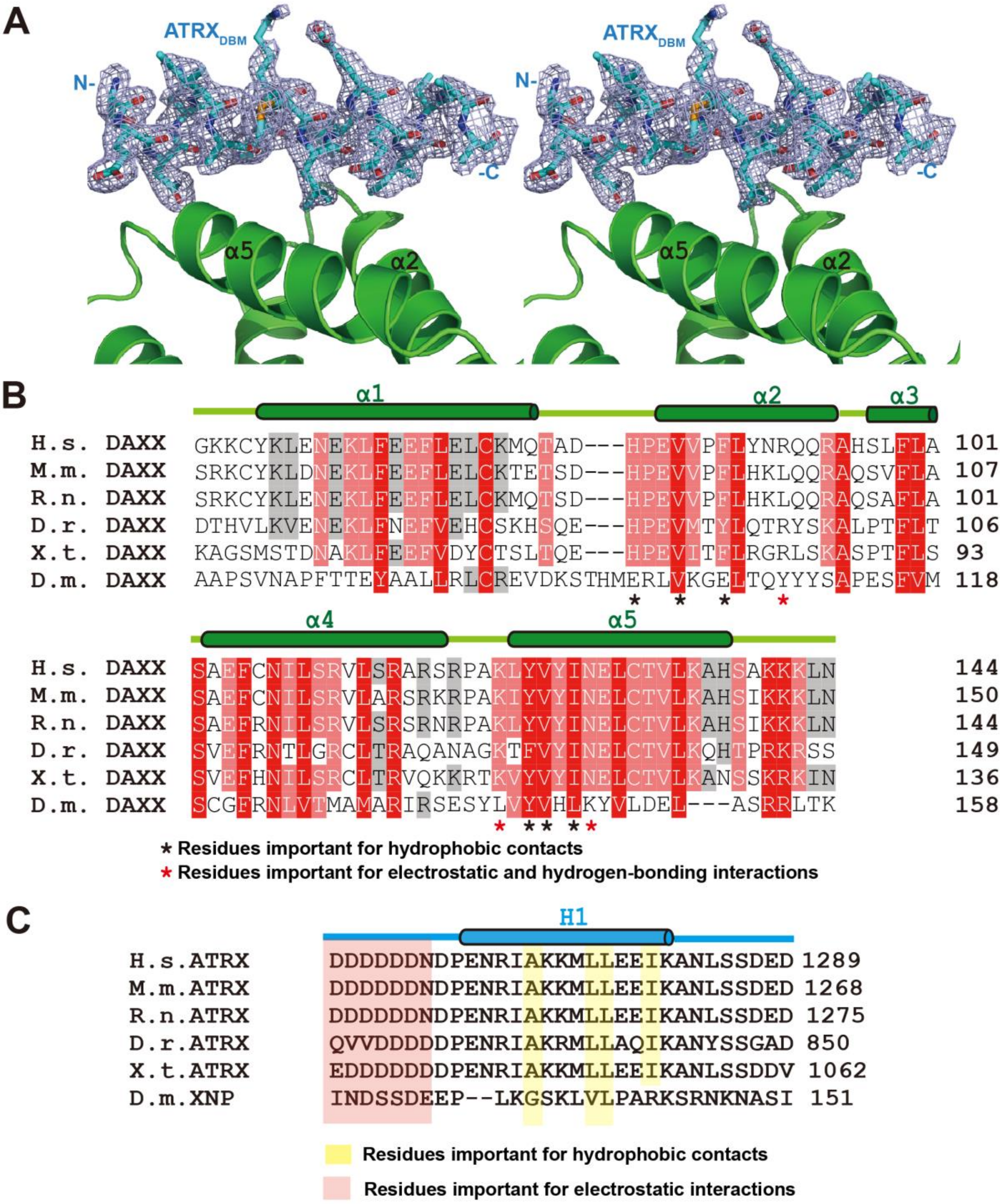

Supplementary Figure 2

Supplementary Figure 2. The crystal structure of $DAXX_{DHB}$-$ATRX_{DBM}$ complex.

A. The $ATRX_{DBM}$-binding-pocket in DAXX shown as stereo images. DAXX is colored in green and ATRX is colored in cyan. The electron density (2Fo-Fc) map, contoured at 1σ, is shown for $ATRX_{DBM}$.

B. Sequence alignment of DAXX proteins from different species. Secondary structure assignments based on the DAXX structure are shown as cylinders (α-helices) and lines (loops) above the sequences. The residues important for hydrophobic contacts and polar interactions are labeled as indicated. H. s., Homo sapiens; M. m., Mus musculus; R. n., Rattus norvegicus; D. r., Danio rerio; X. t., Xenopus tropicalis; D. m., Drosophila melanogaster.

C. Sequence alignment of ATRX proteins from different species. The conserved hydrophobic, aromatic and N-terminal acidic residues important for DAXX-ATRX interactions are labeled.

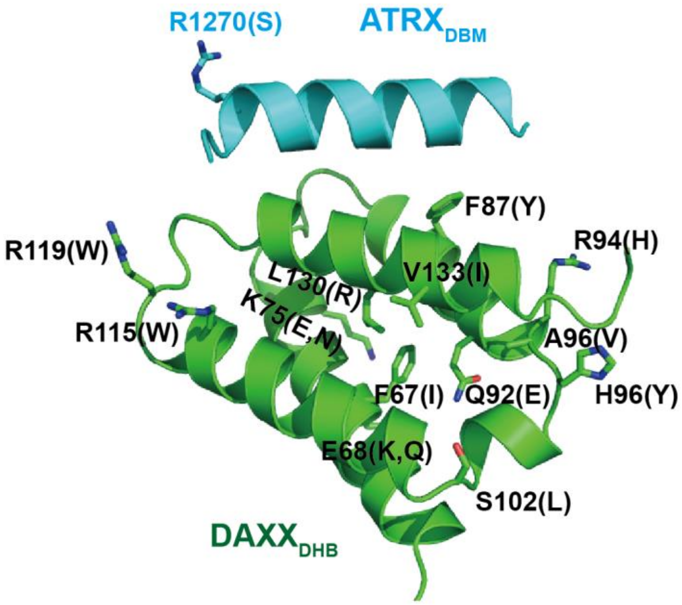

Supplementary Figure 3

Supplementary Figure 3. Disease mutations in the regions of $DAXX_{DHB}$ and $ATRX_{DBM}$.

The mutation information of DAXX and ATRX is from COSMIC (http://cancer.sanger.ac.uk/cosmic). The identified missense mutations are shown as stick models in the structure. The substituted residues are indicated in the parentheses. Most of the residues are critical for protein folding and stability, and are not localized to the interface between DAXX and ATRX, except for DAXX F87. DAXX F87, which is in the interface of DAXX-ATRX, is mutated to Y, and this substitution presumably still maintains the interaction with ATRX.

Supplementary Table 1 Data collection, phasing and refinement statistics

	SeMet $DAXX_{DHB}$-$ATRX_{DBM}$
**Data collection**	
Space group	*C222*$_1$
Cell dimensions	
a, b, c (Å)	69.485, 107.904, 30.887
α, β, γ (°)	90, 90, 90
	Se Peak
Wavelength (Å)	0.97853
Resolution (Å)	50-2.2
R_{merge}	0.077(0.413) *
I / σI	20.3 (4.0)
Completeness (%)	99.6 (99.8)
Redundancy	6.2 (5.4)
Phasing statistics	
No. of Se sites	2
Phasing power (32-2.2Å,SAD)	0.7
R_{cullis} (32-2.2Å,ano)	0.899
Figure of merit (32-2.2Å)	0.17
**Refinement**	
Resolution (Å)	26.98-2.20
No. reflections	6142
No. reflections for R_{free}	311
R_{work} / R_{free}	0.187/0.219
No. atoms	
DAXX	686
ATRX	128
Water	52
B-factors	
DAXX	34.9
ATRX	46.9
Water	45.3
R.m.s deviations	
Bond lengths (Å)	0.002
Bond angles (°)	0.530

*Values in parentheses are for highest-resolution shell.

References

Adams, P.D., Grosse-Kunstleve, R.W., Hung, L.W., Ioerger, T.R., McCoy, A.J., Moriarty, N.W., Read, R.J., Sacchettini, J.C., Sauter, N.K., and Terwilliger, T.C. (2002). PHENIX: building new software for automated crystallographic structure determination. Acta Crystallogr D Biol Crystallogr *58*, 1948-1954.

Emsley, P., and Cowtan, K. (2004). Coot: model-building tools for molecular graphics. Acta Crystallogr D Biol Crystallogr *60*, 2126-2132.

Minor, W., Cymborowski, M., Otwinowski, Z., and Chruszcz, M. (2006). HKL-3000: the integration of data reduction and structure solution--from diffraction images to an initial model in minutes. Acta Crystallogr D Biol Crystallogr *62*, 859-866.

Vonrhein, C., Blanc, E., Roversi, P., and Bricogne, G. (2007). Automated structure solution with autoSHARP. Methods Mol Biol *364*, 215-230.
